# Nanosized Cerium Phosphate: Synthesis Methods, Morphology, and Potential Applications in Biomedicine

**DOI:** 10.3390/biomedicines14061337

**Published:** 2026-06-12

**Authors:** Svetlana A. Titova, Victor A. Stupin, Natalia E. Manturova, Elena L. Chuvilina, Akhmedali A. Gasanov, Vladimir A. Parfenov, Ekaterina V. Silina

**Affiliations:** 1 I.M. Sechenov First Moscow State Medical University (Sechenov University), Ministry of Health of the Russian Federation, 119991 Moscow, Russia; honey.liebe@mail.ru (S.A.T.); chuvilina.elena@lanhit.ru (E.L.C.); vladimirparfenov@mail.ru (V.A.P.); 2 Pirogov Russian National Research Medical University, Ministry of Health of the Russian Federation, 117997 Moscow, Russia; stvictor@bk.ru (V.A.S.); manturovanatali@yandex.ru (N.E.M.); akhmedali@lanhit.ru (A.A.G.)

**Keywords:** cerium phosphate, nanoparticles, nanomaterials, nanotubes, nanorods, nanoceria, synthesis, polymers, UV filter, regenerative effect, redox activity, antioxidants

## Abstract

The growing field of nanobiotechnology could provide an alternative platform for the development of new therapeutic agents. A potential means for achieving these goals are nanoparticles of rare-earth metals, for example, nanoceria. According to the results of numerous in vitro and in vivo studies, not only oxide forms of lanthanides can demonstrate a pharmacological effect. A promising nano-object for biomedical application is cerium phosphate, which exhibits both properties characteristic of cerium dioxide and its own unique properties, due to the diversity of morphology. However, at present, a unified methodological approach has not been formulated that would make it possible to formulate principles for obtaining a compound with specified properties. This review was conducted on using the international databases PubMed, PubChem, Scopus and Google Scholar, and included original studies and reviews. The literature describes the preparation of cerium phosphate nanoparticles by the hydrothermal, chemical precipitation, microwave, and sol–gel methods. It was established that reaction temperature, pH value of the medium, use of organic solvents, ratio of reagents, and precursors have a direct influence on the size, shape, and structure of the obtained nano-object, making it possible to synthesize nanospheres, nanorods, and nanoneedles by regulating these parameters. In addition, the strategy of obtaining nano-objects with specified properties can be implemented by using excipients of predominantly polymer nature. The use of auxiliary substances is capable both of exerting a stabilizing effect and improving adherence to the nanoscale range, and of influencing pharmacological activity. The literature describes the possibility of using cerium phosphate as a redox-active, regenerative, antibacterial, sunscreen, and antitumor agent. However, the insufficient amount of data on the toxicological profile, as well as the results of in vivo studies, remains a significant limitation for the introduction of cerium phosphate into clinical practice. Thus, the purpose of the present review is to identify patterns that make it possible to formulate recommendations for the synthesis of cerium phosphate with specified properties, to assess factors affecting its suitability for use in biomedicine, and to consider its prospects and limitations.

## 1. Introduction

In recent years, the global scientific community has focused on solving fundamental challenges in international healthcare. In particular, the development mechanisms of numerous pathological conditions involve the generation of reactive oxygen species (ROS), which induce oxidative stress [1,2,3]. Oxidative stress exerts a non-selective toxic effect on human organs and tissues, leading not only to localized cellular damage such as ferroptosis, apoptosis, and pyroptosis [4], but also to telomere shortening and dysfunction, resulting in genomic destabilization [5]. This pathogenic factor is a structural component of endocrine [6,7,8], oncological [9,10,11,12], cardiological [13,14], autoimmune [15,16,17], and neurodegenerative diseases [18,19,20], and likely plays a role in reproductive dysfunction [21,22,23]. Many of these disorders impose a significant economic burden [24,25,26,27], reduce patients’ quality of life [28], and lead to social maladaptation [29,30] and disability [31]. Depending on the target of therapeutic intervention, several strategies may be used.

For chronic pathologies associated with a high risk of complications, approaches aimed at correcting risk-modifying conditions may be effective, allowing preventive measures to be taken at early stages. Thus, trophic disorders and infectious complications pose the greatest threat to patients with impaired immune function and reduced regenerative capacity, including bedridden patients [32], elderly individuals with dementia [33], patients with coagulation disorders and deep vein thrombosis of the lower extremities [34], and patients with diabetes mellitus [35,36]. According to Frey C.B. et al., timely initiation of comprehensive treatment in vulnerable patient groups is a prognostically favorable factor and may lead to remission [37]. Therefore, despite the impossibility of radical cure, this line of work represents an important stage in the improvement of medical care.

At the same time, modern medicine pays attention not only to maintaining patients’ quality of life, but is also oriented toward preventing the development of diseases [38,39,40,41]. Advances in modern preventive medicine have made it possible to identify the main risk factors and, consequently, have provided an opportunity to significantly influence the incidence and prognosis of many life-threatening diseases, including cervical cancer [42], hepatocellular carcinoma [43], and colorectal cancer [44]. A reduction in morbidity has been achieved not only through screening programs, but also through the implementation of vaccination programs and informing the population about the necessity of adhering to a healthy lifestyle [45,46]. Despite significant progress in this area, for a number of oncological nosologies, disease prevention remains a significant challenge despite the known risk factors. Melanoma may be cited as an example—a malignant melanocytic neoplasm, one of the main risk factors for the development of which is excessive insolation and, as a consequence, the occurrence of burns and ultraviolet-induced genomic damage to cells [47,48]. Despite the expansion of the range of therapeutic options (for example, the active development of immunotherapy and targeted therapy, as well as research into experimental treatment methods) [48,49,50], the prognosis for many patients remains unfavorable [48,51], which emphasizes the need for further development and dissemination of preventive measures for this disease.

Based on the above, it can be concluded that the challenges facing the modern scientific and medical community collectively form several common directions in pharmaceutical development. Thus, relevant vectors include the creation of agents possessing antioxidant, regenerative, cytoprotective, antitumor, and antibacterial properties. Nanoparticles of rare-earth metals possess these characteristics to varying degrees [52,53,54]. At present, the most extensively studied are cerium dioxide nanoparticles, the pharmacological effects of which have been confirmed in numerous in vitro and in vivo experiments [55,56,57,58]. Taking into account the research experience with other lanthanide nanoparticles (such as lanthanum phosphate and gadolinium vanadate), it may be assumed that not only oxides but also other rare-earth metal compounds could be considered for biomedical applications [52,59]. Based on current evidence, nanosized cerium phosphate represents a promising candidate. It possesses many properties similar to cerium dioxide (such as antioxidant and regenerative activity) [60,61], while also exhibiting specific characteristics associated with its phosphate form, including diverse morphology [62,63].

Therefore, the aim of this review is to provide a comprehensive analysis of cerium phosphate as a nanomaterial, including its properties, synthesis methods, pharmacological effects, and biomedical application prospects.

We provide a comprehensive and critical review of the latest progress in this field. This literature review was conducted using PubMed, PubChem, Scopus and Google Scholar databases. The following keywords were used in preparation of this review: “cerium phosphate” and “nano”, “cerium phosphate” and “nanoneedle”, “cerium phosphate” and “nanorod”, “cerium phosphate” and “nanospheres”, “cerium phosphate “synthesis”, “cerium phosphate” and “hydrothermal”, “cerium phosphate” and “microwave”, “cerium phosphate” and “sol-gel”, “cerium phosphate” and “chemical precipitation”, “cerium phosphate” and “green synthesis”, “cerium phosphate” and “polymer”, “cerium phosphate” and “polyvinylpyrrolidone”, “cerium phosphate” and “polyethylene glycol”, “cerium phosphate” and “polymethacrylic”, “cerium phosphate” and “citric acid”, “cerium phosphate” and “cetyltrimethylammonium bromide”, “cerium phosphate” and “antibacterial”, “cerium phosphate” and “redox”, “cerium phosphate” and “antioxidant”, “cerium phosphate and “pro-oxidant”, “cerium phosphate” and “regenerative”, “cerium phosphate” and “antitumor”, “cerium phosphate” and “sunscreen”, “cerium phosphate” and “SPF”, “cerium phosphate” and “toxicity”. This review analyzed publications on nanosized cerium phosphate from 2003 to 2025. The primary inclusion criteria were data validity and relevance to the research objectives. The exclusion criteria were based on an analysis of compositions containing cerium phosphate that clearly do not meet the definition of “nanosized” (>100 nm in diameter and length). We did not provide an assessment of the risk of bias, and this review was not registered or presented as a systematic review.

## 2. Synthesis Methods

Several techniques are currently used to synthesize nanosized cerium phosphate, each with methodological features that directly determine its suitability for biomedical applications. The literature describes microemulsion synthesis [64], various “green synthesis” approaches [65], and others. However, four methods are the most widely used for producing cerium-containing nanomaterials: (1) hydrothermal synthesis [66], (2) chemical precipitation (coprecipitation) [61,67], (3) microwave synthesis [68], (4) sol–gel method [69].

### 2.1. Hydrothermal Synthesis

**Figure 1:**
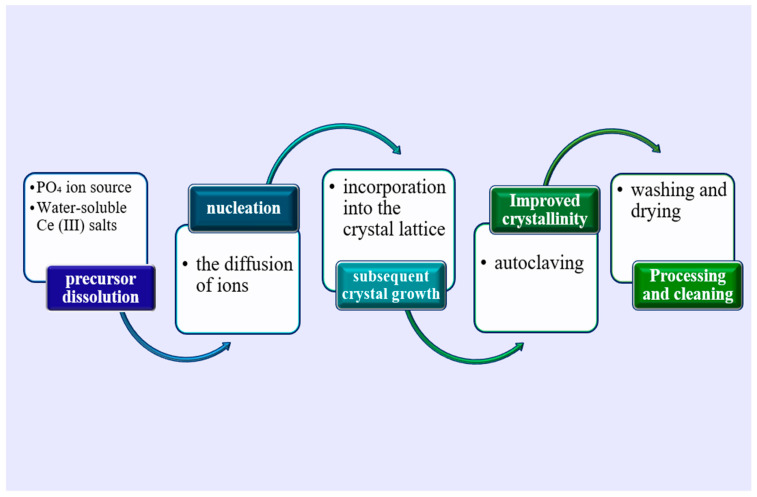
Schematic representation of the hydrothermal synthesis of cerium phosphate nanoparticles.

Hydrothermal synthesis is one of the most studied and widely used methods for producing nanomaterials, including cerium-containing nanoparticles [70]. The general scheme of the method involves precursor dissolution, nucleation, and subsequent crystal growth at elevated temperatures (Figure 1).

In this method, precise control of reaction parameters is crucial to obtain nanoparticles with optimal aggregative and sedimentation stability [71].

Water-soluble cerium(III) salts, such as Ce(NO_3_)_3_ [72,73] or CeCl_3_ [71], in combination with a phosphate ion source, are used as precursors to obtain a supersaturated solution. Traditionally, (NH_4_)_2_HPO_4_ [73] or H_3_PO_4_ [72] are used as phosphate ion donors; however, in some studies, the use of 0.001M Na_3_PO_4_ has also been described [71]. Crystal growth is determined by the diffusion of ions to the surface of the growing crystal and their subsequent incorporation into the crystal lattice. The ratio of these precursors [72], as well as the pH of the reaction medium [71,73], has a significant influence on morphology. At the same time, it is noteworthy that the choice of solvent does not have a substantial effect and demonstrates similar particle sizes (for example, when using water [71] and N,N-dimethylformamide [74], the particle diameter differed by only 10 nm). However, the most critical synthesis parameters with respect to particle size are temperature and reaction time.

Thus, higher temperatures generally lead to higher crystallinity. At the same time, objects obtained at higher temperatures, as a rule, have larger particle sizes, and although their diameters remain within the concept of “nanoscale” (5–50 nm), the overall length may reach several micrometers [71]. At a temperature of 100 °C, nano-objects with predominantly hexagonal morphology are formed. When heated from 180 °C, monoclinic objects begin to be detected, which become predominant upon reaching 200 °C [71]. At the same time, the literature also describes methods for obtaining monoclinic structures at lower temperatures through regulation of component ratios, which emphasizes the role of controlling a combination of factors during synthesis [72].

After synthesis, CePO_4_ nanoparticles are usually subjected to washing and double drying (with the first autoclaving at a temperature of 90–100 °C [71,73]) in order to remove residual precursors and solvents. The literature describes triple washing with distilled water and absolute ethanol, followed by vacuum drying with heating up to 60 °C [71,73]. In this case, the ethanol-to-water ratio may vary over a wide range, for example from 1/35 to 21/15 [73]. To prevent oxidation of synthesis intermediates, the use of an inert gas, argon, is possible [72]. In turn, the reaction duration (which varies most significantly at this stage) exerts a specific effect on cerium phosphate nanoparticles. According to data reported by Yang M. et al., luminescence ability is increased in samples obtained during prolonged reactions. The researchers explain this phenomenon by the fact that the ordered “bundle-like” (or “flower-like”) structures obtained by them possess fewer surface defects and are less contaminated, which is confirmed by the results of X-ray photoelectron spectroscopy [73].

Thus, it can be concluded that hydrothermal synthesis makes it possible to regulate the size and morphology of nano-objects by modifying synthesis conditions. Using this synthesis method, predominantly nanowires and nanorods are obtained, often hierarchically organized into spherical structures. At the same time, despite the optimal rod diameter (approximately 5–50 nm), the rod length may be potentially excessive for biomedical applications. In addition, a significant limitation of this method is the use of chemically aggressive reagents (such as nitric or hydrochloric acid), the need to apply high temperatures, and the extremely long synthesis duration. At the same time, it is not possible to avoid these negative factors, since they exert a critical influence on particle characteristics (in particular, aggregative stability and particle size). The possibility of influencing these factors through the use of auxiliary substances or doping is also associated with limitations, since surface modification requires the formation of monoclinic structures [75], which, as mentioned earlier, require achieving a temperature of at least 100 °C. Based on this, the question of the feasibility of using cerium phosphate obtained by the hydrothermal method remains debatable.

### 2.2. Chemical Precipitation

Chemical precipitation is a widely used method for the synthesis of cerium phosphate (CePO_4_) nanoparticles, due to its simplicity, scalability, and the possibility of controlling the size and morphology of the final products [61,76]. The method is based on mixing aqueous solutions containing cerium cations (Ce^3+^) and phosphate anions (PO_4_^3−^), which leads to the ultrafast formation of an insoluble CePO_4_ precipitate. The fundamental scheme of physicochemical processes (crystal formation, ion diffusion, etc.) is similar to that of the hydrothermal method [77].

In this method, Ce(NO_3_)_3_·6H_2_O and NH_4_H_2_PO_4_ are predominantly used as precursors. A distinctive feature of the approach is the possibility of operating under alkaline pH conditions through the addition of ammonium hydroxide intended for precipitation [76,78]. At the same time, it should be noted that alkalization is not a mandatory condition, and the reaction is possible at a pH of approximately 2 [61]. This method requires prolonged stirring of the reaction mixture (approximately half an hour) under heating. The calcination temperature exceeds that of the hydrothermal synthesis method and may reach 800 °C. This is due to the fact that, for this method, the relationship between temperature and the predominant form of the objects—hexagonal or monoclinic—also remains valid [76]. As a result, it is possible to obtain porous structures with spherical morphology, the size of which does not exceed the diameter of objects obtained by the hydrothermal method and, accordingly, is several orders of magnitude smaller than the length of nanorods and nanowires [61,76]. At the same time, in the case of the formation of anisotropic needle-like particles, their dimensions also fully corresponded to the nanoscale range and reached up to 50 nm in the largest dimension. Despite this, the authors of the study noted particle agglomeration when analyzed using transmission electron microscopy [61]. X-ray diffraction analysis, as well as emission assessment conducted by Verma S. et al., confirmed high purity, the absence of pronounced defects, and the activity of the nanospheres [76], which makes cerium phosphate obtained by this method similar in many parameters to cerium dioxide and, consequently, indicates its significance for further research. The key advantage of this method is the achievement of nanosized particles (<100 nm in diameter and length) with a porous structure. At the same time the morphology of the objects is heterogeneous and is represented by both nanoneedles and nanospheres.

Another significant factor is that this method also requires the use of high temperatures, as well as considerable time expenditures. An additional stirring stage may also have a negative impact when selecting an appropriate technological process scheme in attempts to scale up production. At the same time, the chemical precipitation method can probably be considered optimal for synthesis under well-equipped laboratory conditions and on a small scale.

### 2.3. Microwave Synthesis of Cerium Phosphate Nanoparticles

Microwave synthesis is a promising method for obtaining cerium phosphate (CePO_4_) nanoparticles, offering significant advantages compared to traditional methods such as chemical precipitation or hydrothermal synthesis. Strictly speaking, microwave synthesis is considered primarily as a modification of the hydrothermal method, with a number of differences. This method is based on the ability of dielectric materials to absorb microwave radiation energy and convert it into heat, ensuring rapid and uniform heating of the reaction mixture during synthesis. The method uses microwave radiation energy to accelerate chemical reactions, which leads to a reduction in synthesis time, improvement in particle homogeneity, and more efficient control over their size and morphology [79].

In a typical microwave synthesis, cerium precursors (for example, Ce(NO_3_)_3_) and phosphate precursors (for example, Na_3_PO_4_) are dispersed in purified water [68], after which the solution is subjected to microwave irradiation for a specified period of time. In the literature, synthesis with radiation power ranging from 180 W [68] to 200 W [79] is the most common. The duration of microwave exposure usually varies from 15–30 min to one hour, which surpasses even synthesis methods using ultrasound [68,79,80].

For this method, the use of an acidic reaction medium is also typical. An interesting feature is the possibility of operating within a relatively wide range of pH values (from 1 to 5), provided by the addition of nitric or phosphoric acid [79]. It should be noted that under these conditions, monoclinic nanorods with an acceptable diameter (approximately 50 nm), but with a length of up to 2–3 µm, are formed, which imposes limitations on application possibilities [68]. It is also possible to obtain nearly spherical particles prone to aggregation [79].

Summarizing, the microwave method makes it possible to somewhat accelerate the synthesis process and, probably, due to this not only reduce labor costs but also decrease the likelihood of the formation of any by-products. In turn, the disadvantages include the need for specific equipment, which is expensive relative to other methods.

### 2.4. Sol–Gel Method

**Figure 2:**
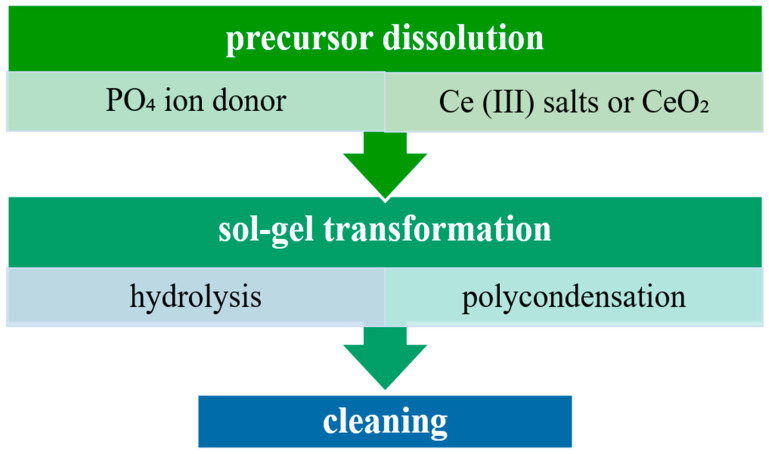
Schematic representation of sol–gel synthesis of cerium phosphate nanoparticles/nanofibers.

Sol–gel technology represents a multistage chemical process based on the formation of a colloidal solution from molecular precursors, followed by its transformation into a three-dimensional network structure through hydrolysis and polycondensation reactions (Figure 2).

At present, this method for obtaining nanosized cerium phosphate is less extensively described in the literature than the others and is largely experimental in nature. Thus, a non-standard approach was described that involved the use of cerium dioxide as a cerium source, which was dispersed in an acid. The phosphate ion donor in this work remained classical-phosphoric acid was chosen, and the cerium-to-phosphate anion ratio was 1:140. A distinctive feature of the method is the addition of a number of reagents that are not commonly used in other chemical strategies, such as methyl tert-butyl ether. A specific feature of the product obtained by Yorov K.E. et al. is the long gel “aging” period—over 10 days, followed by replacement of the solvent with acetonitrile and subsequent supercritical drying in CO_2_. The result of this process was the production of an aerogel that was practically ready for use and contained cerium nanofibers [69].

Another example, somewhat more consistent with classical approaches, is the synthesis described in the study by Llusar M. et al. In this work, standard precursors were used. A notable feature of the experiment is the use of a pH-sensitive organic component, N-dodecanoylglycyl-L-alanine, as well as the high synthesis rate; when obtained without a template, the duration was approximately 120 h. At the same time, a limitation is the requirement for calcination under harsh conditions. The fibers described had significant dimensions: width up to 100 nm, thickness up to 140 nm, and length up to hundreds of micrometers [81]. The effectiveness of such samples for therapeutic application is currently unknown. Presumably, the lipopeptide nature of the excipient ensures high biocompatibility; however, the bioavailability of cerium in this form requires further investigation.

In contrast, a single-stage method for obtaining a gel containing exclusively cerium phosphate was described. The aerogel was obtained without the use of organic gelling agents. The cerium source in the experiment was also pre-synthesized cerium dioxide, while inorganic acids were selected as additional components. Supercritical drying was identified as the key stage of the process. According to Kozlova T. et al., the key factor in obtaining a cerium-containing gel is the intrinsic ability of cerium phosphate to bind water (up to 200,000 water molecules per cerium atom). The main limitation of this approach was that the gels were destroyed during aging if the cerium phosphate-to-water ratio exceeded 1:6 [82]. For comparison, according to the method of Yorov K.E. et al., gel formation, on the contrary, did not occur when the cerium phosphate-to-water ratio was less than 1:1060 [69]. Thus, the single-phase method leads to the formation of sufficiently highly concentrated compositions, which may both increase efficiency and increase the risk of toxic and local irritant reactions.

### 2.5. Green Synthesis

In recent years, green synthesis has become an increasingly popular approach for the production of nanomaterials within the international scientific community. This is primarily due to the possibility of performing synthesis without the use of highly toxic and chemically aggressive agents and, consequently, without causing harm to the environment [83]. In addition, significant advantages of this method include a wide scope for experimentation and new discoveries, owing to the broad range of biological systems that can be employed.

For oxide nanoparticles there exists an extensive range of green synthesis strategies demonstrating relevant results. An example is the synthesis of nanoceria using such unconventional components as extracts of *Cucurbita pepo* [84], *Acorus calamus* [85], or *Chenopodium quinoa* L. [86]. In turn, the synthesis of nanoscale cerium phosphate via green methods has its own specific features.

Thus, in the study by Pusztai P. et al. [65], the synthesis of nanowires and nanoneedles is described using standard precursors (phosphoric acid and cerium III nitrate hexahydrate in a ratio of 1.1 mL of 85% acid diluted to 80 mL and 240 mL of a 0.03 mM cerium salt solution). The synthesis time ranged from almost instantaneous product formation to 3 h. At the same time, the authors did not employ auxiliary reagents (except for the preparation of terbium-doped derivatives, where terbium was used to regulate the transition from a hexagonal to a monoclinic structure) or extreme physical treatments. This distinguishes the approach from other strategies. At the same time the authors highlighted a number of challenges associated with this method. In particular, the high reaction rate made it difficult to monitor the formation of nanostructures and maintaining the process required dynamic regulation of the pH of the reaction medium, which increased the labor intensity of the process [65].

In contemporary literature, greater attention is given to the synthesis of nanocomposites containing cerium phosphate via green methods. In particular, the preparation of composites consisting of hexagonal La_2_O_3_ and monoclinic CePO_4_ using an extract of the soft coral *Sinularia polydactyla* has been described. Lanthanum III nitrate hexahydrate and cerium III nitrate hexahydrate were used as precursors and the pH was adjusted by the addition of 0.1 M hydrochloric acid. The average particle size was 29.81 nm, and the obtained composites did not exhibit cytotoxicity in studies with HeLa cervical cancer cell cultures [87].

In addition, studies have been reported on the synthesis of CeO_2_-CePO_4_ nanocomposites using a method combining the sol–gel process and green synthesis. The composite was obtained using a 0.1 M solution of Ce(NO_3_)_3_·6H_2_O as a precursor, with *Artocarpus heterophyllus* extract serving as both a reducing agent and a source of phosphate ions, followed by calcination at 500–900 °C. These composites also did not demonstrate cytotoxicity toward HeLa cell cultures, as well as Vero cells (African green monkey kidney epithelial cells) [88].

Particular attention in this review should be given to a study that does not directly address the synthesis of nanomaterials but potentially opens a new direction for research. In the work by Kang X. et al., the ability of spent culture liquid of *Aspergillus niger* and struvite filtrate to extract various cerium derivatives from solution is described. It was shown that at a CeCl_3_ concentration of 20 mM in the medium, the formation of highly purified nanoparticle aggregates was observed, with an almost 100% yield of cerium phosphate after thermal treatment at 1000 °C. Based on these results, it can be assumed that natural systems may be used as a tool for the synthesis of nanoscale cerium phosphate alongside cerium dioxide. At the same time, despite the promising prospects for further research, a significant limitation was identified: when the CeCl_3_ concentration was increased to 50 mM, the yield of cerium phosphate decreased to 90.4% [89].

Thus, in this section, the key features of methods for the synthesis of nanosized cerium phosphate were considered, ranging from those that have long become traditional in global chemical science to a number of experimental techniques. The main characteristics of the processes are highlighted and summarized in Table 1.

Summarizing this section, we can conclude that the most promising method for further research is chemical precipitation, which makes it possible to obtain smaller particles that are more often prone to isotropy. At the same time, a more predictable morphology, albeit with a larger particle size (more precisely, fibers or rods), is achievable for the hydrothermal method and its modifications, which allow the process to be somewhat accelerated and reduce the likelihood of obtaining by-products. At the same time, the sol–gel transition method, which is poorly studied specifically for cerium phosphate, may be of great interest to researchers, since, despite the significant labor intensity of existing techniques, as well as the absence of clearly formulated recommendations for carrying out the synthesis process, it is this method that may become the main step toward obtaining agents for biomedical application. The key factors for the development of this method may be studies of pharmacological and toxic effects on cell and tissue cultures and, possibly, subsequently, in vivo.

It should be noted that in many of the aforementioned studies, the synthesis products extend beyond the nanoscale range. However, they may still be of interest to researchers working with micro-objects. At the same time, a significant limitation remains the lack of data on the zeta potential of the obtained materials. This is likely due to the fact that these materials were not originally intended for biomedical applications, and therefore the assessment of parameters characterizing the stability and aggregation stability of the synthesis products was not undertaken.

## 3. Morphology of Cerium Phosphate

**Figure 3:**
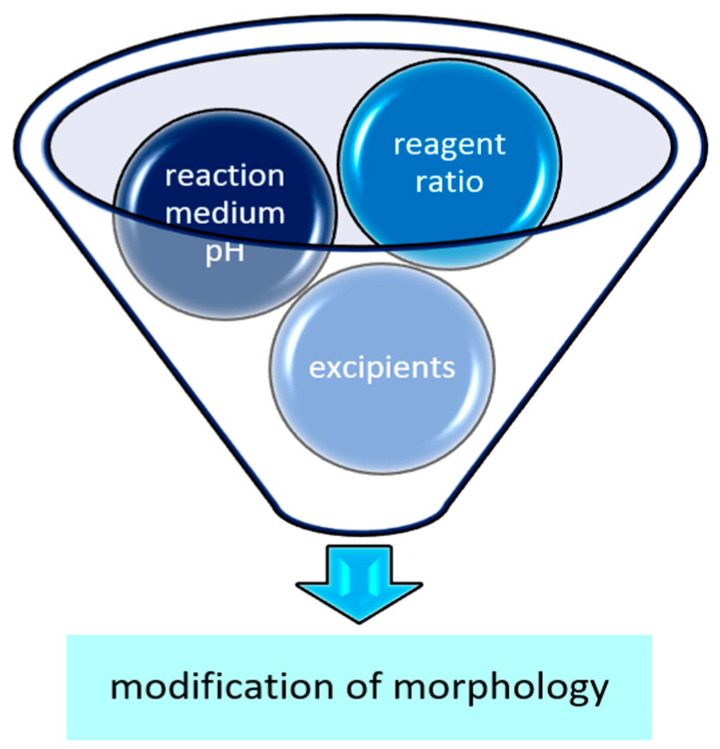
Additional factors influencing the morphology of nanosized cerium phosphate.

In addition to the size of individual particles, the practically unique morphological characteristics of cerium phosphate play a significant role. The morphology of nanosized cerium phosphate (CePO_4_) determines its physicochemical properties. This is mainly related to the relationship between particle morphology (e.g., nanospheres, nanorods, etc.) and structural anisotropy. In addition to the synthesis method, the CePO_4_ nanoparticle morphology is also influenced by the reagent ratio and the pH of the solution used during the fabrication process [72,76,90] (Figure 3).

### 3.1. Influence of Reagent Ratio on the Morphology of Nanosized Cerium Phosphate

The ratio of reagents is one of the determining factors controlling the morphology of nanosized cerium phosphate (CePO_4_). As an example, let us consider the hydrothermal synthesis of CePO_4_ using cerium(III) nitrate and orthophosphoric acid (H_3_PO_4_) as precursors. Bao J. et al. conducted an experiment in which the researchers performed a comparative analysis in the range of molar concentration ratios from 1:10 to 1:600. According to the researchers, at a low reagent ratio, rod-shaped structures are formed. When the ratio is increased to 1:120, these rods self-organize into complex structures resembling bundles. A further increase in the difference between the precursors affects only the degree of binding of phosphate tetrahedra without a fundamental change in the shape of the obtained nano-objects. At the same time, it should be noted that with an increase in the ratio, a significant increase in rod length (up to 1 μm) was observed, which undoubtedly goes beyond the concept of “nanoscale” and may lead to a decrease or complete loss of pharmacological effect [72].

Another example is chemical precipitation, in which the optimal molar ratio of precursors is 1:1, as a result of which homogeneous spherical structures are formed [76], which emphasizes the need for individual selection of parameters for each method.

### 3.2. Influence of Reaction Medium pH

The morphology of nanosized cerium phosphate (CePO_4_) demonstrates a pronounced dependence on the pH of the synthesis medium, which is due to the complex interaction of cerium ions and phosphate ions in solution. Variations in pH have a significant effect on the processes of nucleation, growth, and agglomeration of CePO_4_ nanocrystals, determining the final shape and size of the obtained particles [90].

In an acidic medium (pH from 1 to 5 [68]), the tendency toward the formation of increased concentration of protons (H^+^) promotes protonation of phosphate ions (HPO_4_^2−^, H_2_PO_4_^−^), reducing their reactivity toward cerium ions (Ce^3+^) [90]. As a result, the nucleation rate slows down, which leads to the formation of a smaller number of crystallization centers and, accordingly, to the formation of smaller particles. For example, during the synthesis of CePO_4_ using cerium nitrate (Ce(NO_3_)_3_) with sequential addition of nitric acid, nanorods are formed, which is due to anisotropic growth of CePO_4_ crystals along a certain crystallographic axis. When the amount of acid added dropwise to the reaction mixture was increased from 3 mL to 3.8 mL, the absence of formation of large spherical structures formed from rods was noted, which can be interpreted as a significant decrease in the tendency toward aggregation [73]. Another example of obtaining cerium phosphate nanoparticles in an acidic medium is the use of 0.1 M hydrochloric acid to a pH value < 1. In this method, the rate of ion movement reaches maximum values and, as a consequence, morphologically uniform hexagonal nanowires are formed [71]. At the same time, it should be noted that rods are reproducibly obtained at the lowest values (pH from 1 to 2), while when these values are exceeded, particle aggregates arise [68].

In an alkaline medium (pH about 11–12), deprotonation of phosphate ions (PO_4_^3−^) occurs, which increases their reactivity and promotes faster nucleation and growth of CePO_4_ crystals. When CePO_4_ is precipitated from solutions containing ammonium hydroxide (NH_4_OH), small spherical CePO_4_ particles are formed. In a number of cases, values were obtained indicating the smallest particle diameter when synthesized in this range (for example, 10–17 nm at pH = 11 compared with 15–21 nm at pH = 3) [79]. However, under these conditions, the formation of particles with lower aggregation stability is possible due to the high crystallization rate and insufficient surface stabilization [76]. It is noteworthy that the morphology of particles obtained in an alkaline medium is similar to nanospheres formed in non-aqueous solvents (such as, for example, N,N-dimethylformamide) [74].

Thus, the use of alkaline reagents can provide optimal size of particles; however, such pharmaceutical compositions may require the introduction of additional excipients to ensure stability.

Organic acids, salts, and various polymers can be considered as possible candidates for the role of stabilizers or surface modifiers. Polymeric stabilizers, such as polyvinylpyrrolidone (PVP), act via steric stabilization. Polymer molecules adsorb on the surface of nanoparticles, creating a steric barrier that repels neighboring particles, preventing their agglomeration, which leads to the formation of a stable colloidal dispersion.

Polyvinylpyrrolidone is a nonionic polymer consisting of N-vinyl-2-pyrrolidone units. PVP is a biocompatible polymer that does not cause DNA damage in cell cultures [91], including cultures of normal epithelial cells of the PNT1A type [92], as well as in in vivo studies [93]. Due to this, PVP is used as an excipient in the manufacture of medicinal products [94,95], and is also included in innovative developments for medical use [96]. At the same time, it should be noted that PVP can cause anaphylactic reactions [94], and with prolonged administration of PVP by injection, the development of renal tubular atrophy and chronic kidney disease is possible [97].

PVP has been described as a stabilizer for many nanoparticles [98], including various rare-earth metals [99], and especially cerium dioxide nanoparticles [100,101,102]. It is likely that the possible mechanism of stabilizing action lies in the fact that the nitrogen-containing pyrrolidone group of PVP serves as a ligand that coordinately binds to cerium ions, temporarily blocking access to certain facets of the forming nano-object. This leads to anisotropic crystal growth and the formation of nanostructures with predetermined characteristics. According to researchers, the synthesis of nanoceria using PVP is technically quite simple and makes it possible to obtain particles of small diameter (up to 20 nm) with high physical and chemical stability [103]. At the same time, according to the latest data from international scientific literature, PVP can be used to stabilize nanosized cerium phosphate both in isolated form [70,104] and when doped with other metals [65].

Thus, Cheng C. et al. in 2021 [70] described the production of cerium phosphate nanorods by a hydrothermal method with the addition of polyvinylpyrrolidone prior to synthesis. The obtained sample had a monoclinic structure and high redox activity. It should be noted that in this in vitro study, the size of nano-objects as well as their stability were not assessed, which was due to the researchers’ focus on the electrochemical activity of the product [70].

Of particular interest is the research work published by Römer I. et al. in 2019 [105]. The scientists did not carry out targeted synthesis of cerium phosphate, but studied possible chemical and environmental transformations of cerium dioxide nanoparticles. At the same time, nanoceria (also obtained by a hydrothermal method) was coated with PVP with a molecular weight of 10,000. Potassium dihydrogen phosphate was used as a phosphate ion donor. The study was conducted over a wide range of pH values (strongly acidic, weakly acidic, and alkaline media). When the PVP concentration was increased from 1 mmol to 5 mmol, the formation of phosphate was detected at each hydrogen index value. In this study, the size of the obtained particles was also not evaluated; however, it may be noteworthy in that cerium phosphate was obtained under exceptionally mild conditions. Despite the fact that pH values of 2.3 and 12.3 were used in some experiments, the researchers did not apply temperature or pressure increase, autoclaving, or microwave radiation. Thus, the described observation may become a prerequisite for the development of a fast, environmentally friendly, and economical method for the synthesis of nanosized cerium phosphate [105].

When considering the possibility of using cerium dioxide as a precursor for the synthesis of cerium phosphate nanoparticles, it is necessary to take into account the results of the study by Ta K. et al. [106]. The article described the binding of phosphates on the surface of nanoceria. This was confirmed using such high-precision analytical methods as infrared and Raman spectroscopy [106]. Based on this, it should be concluded that confirmation of the actual transformation of dioxide into phosphate, rather than adsorption, must be reliably supported by assessment of particle morphology and determination of the composition of the interaction product.

In another study devoted to the development of methods for synthesizing nanoparticles of rare-earth metals, the possibility of using PVP with a molecular weight from 40,000 to 360,000 was described. The scientists evaluated the relationship between the molecular weight of polyvinylpyrrolidone, the stirring speed during synthesis, and the morphology of the obtained particles. According to the conclusion of D’Alonzo N.J. et al., at a speed of 3000 revolutions per minute, no differences were observed between particles stabilized with PVP 40,000 and 360,000. Already at a speed of 5000 revolutions per minute, it was found that particles stabilized with PVP 360,000 formed smaller and thinner bundles. More pronounced differences were observed at a speed of 9000 revolutions per minute: particles stabilized with PVP 40,000 formed bundles of nanorods, whereas those coated with PVP 360,000 formed individual nanorods. It should be mentioned that cerium was not the main focus of the study and was only part of a mixture of lanthanum chloride, cerium nitrate, and terbium chloride prepared in a ratio of 9:8:5 [107]. Thus, we once again draw attention to the fact that when developing compositions containing nano-objects, it is necessary to carefully consider not only the synthesis method, the choice of precursors, and the value of the hydrogen index, but also the choice of a specific excipient and each component of the process.

An alternative polymer excipient that can be used to stabilize nanosized cerium phosphate is polyethylene glycol.

The use of polyethylene glycol (PEG) to improve the properties of an active pharmaceutical ingredient is widespread in modern medicine and pharmacy [108]. Thus, at present, such drugs as PEGylated doxorubicin, interferon alpha-2a and alpha-2b, and naloxone are used in clinical practice [109,110,111,112]. Currently, studies are being conducted on the possibility of using PEGylated vancomycin, oral insulin, disulfiram, and paclitaxel [113,114,115,116]. The use of polyethylene glycol as a stabilizer of nanoparticles, such as, for example, nanoparticles of silver, iron, titanium, aluminum, graphene, and copper (predominantly in oxide forms), has also been repeatedly described in international scientific literature [117,118,119,120,121,122]. In addition to maintaining particle stability itself, it has been established that PEGylation is capable of enhancing the pharmacological effect of the active molecule, as well as providing targeted action and, as a result, improving the tolerability of therapy [118,123,124]. Naturally, studies have also been conducted on PEGylation of cerium-containing nano-objects. Undoubtedly, a larger number of studies address the possibility of using PEGylated cerium dioxide. For nanoceria, the role of polyethylene glycol application was described in detail by Majed Alrobaian. According to data from a large-scale review published in 2023, PEGylation contributed to increased tissue permeability without altering the chemical or physical properties of the nanoparticle [125]. Experimental works described the achievement of excellent aggregation stability-obtaining particles with an average diameter of 5 nm [126]. Due to the combination of these factors, interest has arisen in the scientific community in the possibility of combining polyethylene glycol and nanosized cerium phosphate.

Despite the fact that existing publications devoted to this topic do not consider cerium phosphate as an object for biomedical application, their analysis is appropriate from a chemical-pharmaceutical point of view. In particular, in the experimental work by Shiralizadeh Dezfuli A. et al., a method for obtaining cerium phosphate from cerium nitrate and ammonium dihydrogen phosphate (containing polyethylene glycol) in a molar ratio of 1:1 at pH = 2 using ultrasound for 22 min was described. Samples obtained by this method were characterized by the formation of soft hexagonal “coral-like” structures. According to the authors, this can be explained not only by the fact that against the background of cavitation caused by ultrasound, spherical particles are transferred to the surface of already formed ones, but also by the influence of the functional hydroxyl group of polyethylene glycol. This was confirmed by a series of experiments with ethanol, which also contains a hydroxyl group. These samples, as well as nanoparticles and nanorods of cerium phosphate, demonstrated the ability to fluoresce and were evaluated as a means for detecting lead ions in an aqueous environment. Among the limitations of this study are the absence of an assessment of the size of the obtained objects, as well as the indication of the molecular weight of the polyethylene glycol used. Despite this, the work makes a significant contribution to the development of accelerated synthesis methods and may also be of considerable interest to specialists in the fields of toxicology, diagnostics, and environmental sciences [90].

Another example of the use of polyethylene glycol in the production of nanosized cerium phosphate, including doped forms, is the original article by Kouass S. et al. It considered the use of polyethylene glycol with a molecular weight of 6000. The synthesis was carried out by a hydrothermal method using precursors similar to those described earlier, with drying at a temperature of 150 °C. Cadmium and a combination of cadmium and lithium were selected as doping elements. The structure of the cerium phosphate particles was also hexagonal, but represented by nanorods. The obtained samples possessed high electrochemical activity, with the most pronounced effect for products containing lithium [127]. Thus, the possibility of not only stabilization but also doping should be carefully studied when selecting the optimal formulation for a pharmaceutical composition. At the same time, the possibility of using nanosized cerium phosphate with polyethylene glycol should be subjected to thorough evaluation by conducting studies establishing the size of nano-objects, long-term stability, and across a wide range of molecular weights before its application in pharmaceutical in vitro experiments can be considered appropriate.

In addition to classical polymers that have long proven themselves in pharmaceutical practice, it is possible to consider relatively new and as yet little-known excipients for the stabilization of nano-objects. An example of such excipients are poloxamers (also known as pluronic). They are nonionic stimulus-sensitive biocompatible triblock copolymers characterized by relatively low tissue adhesion and a high ability for functionalization and formation of various structures-from nanospheres to hydrogels [128,129,130]. The monomer units of poloxamers consist of hydrophobic polypropylene oxide chains located between two hydrophilic polyethylene oxide fragments [131].

The literature describes the ability of poloxamers to provide highly effective targeted prolonged therapeutic action of an effector molecule and to reduce the risk of adverse reactions [132,133,134,135]. From a technological point of view, poloxamers possess properties not only of a gel capable of incorporating active ingredients, but also of a surfactant [136]. However, the role of poloxamers is not limited only to optimal properties as excipients in pharmaceutical development. According to the results of numerous studies, poloxamers can find application as components of regenerative and reconstructive agents [137,138,139]. According to a large-scale review published in 2023, possessing a high elastic modulus, stability, and biodegradability, poloxamers are also capable of providing optimal moisture of the wound surface and, depending on the specific modification, demonstrating the ability for self-fixation, which may be useful for preventing the development of postoperative adhesions or may find application as an alternative to suturing certain wounds [140]. In addition, during in vitro, ex vivo, and in vivo experiments, it was confirmed that poloxamers are capable of exhibiting properties of tissue permeability enhancers as well as maintaining antioxidant activity [141]. This synthetic polymer has also been studied in combination with various metal nanoparticles [142,143], including cerium, for which the preservation of the ability to neutralize reactive oxygen species was also confirmed [144].

The combination of poloxamers and cerium phosphate is also discussed in international scientific literature. In published experiments, the possibility of using Pluronic^®^ P123 was described. According to the method of Bu W. et al., the poloxamer was added to the synthesis intermediate during stirring and heating to 35–40 °C, after which autoclaving at 100 °C was carried out. The diameter of the obtained nanorods was about 10–12 nanometers, while the length reached several hundred nanometers. At the same time, rods synthesized without the use of Pluronic^®^ demonstrated significantly greater size variability. According to the researchers’ conclusion, it was the poloxamer that ensured uniformity of nanorod sizes, clarity of facets, and the absence of surface defects, which contributed to an increase in the photoluminescence ability of the objects [145].

In addition to classical nano-objects, the scientific literature describes the possibility of obtaining so-called heterogeneous “nanocables”, crystalline structures containing cerium phosphates, lanthanum, and terbium ions. The authors of the study described a classical hydrothermal method with the creation of pH = 1 by adding 0.1 M hydrochloric acid. Before the autoclaving stage at 100 °C, Pluronic^®^ P123 was introduced into the reaction mixture. According to X-ray diffraction analysis data presented by the authors, the result of such synthesis was hexagonal cerium phosphate cores coated with lanthanum. It is noteworthy that in this composition the particle size of cerium phosphate was 10 nanometers, and the presence of an additional coating increased the diameter only twofold, which still represents an optimal value for nano-objects. In addition, these “nanocables” demonstrated high luminescent activity, which makes them potentially promising agents for the development of diagnostic tools [146].

Despite the numerous advantages described for the application of nanosized cerium phosphate synthesis in combination with poloxamer, some studies demonstrate potential limitations for the use of this method for the purpose of developing agents for biomedical application. In particular, during the study of luminescence ability, the absence of bands characteristic of Ce^3+^ in the ultraviolet region was noted [147]. Based on this, we can assume that cerium phosphate synthesized in this way may not only fail to exhibit antioxidant properties, but may also exert a pro-oxidant effect, which narrows the possible spectrum of use and increases the likelihood of the development of undesirable reactions. Based on this, we can conclude that for this pharmaceutical formulation it is advisable to conduct an additional assessment of redox activity, as well as, probably, to consider these nanorods in the context of antibacterial and antitumor agents, but not regenerative medical products.

In addition to the wide range of nonionic excipients potentially suitable for use in formulations with nanosized cerium phosphate, it is also necessary to consider ionic polymers, a prominent example of which is polymethacrylic acid (PMAA).

Polymethacrylic acid is currently considered a promising excipient in pharmaceutical development. This is due to the fact that this biocompatible polymer exhibits pronounced pH sensitivity, which manifests itself in conformational changes and, as a consequence, changes in the release rate of the active pharmaceutical substance loaded into the polymethacrylate [148,149]. Thus, the release rate of the active substance at pH below 6.8 significantly exceeds that at pH = 7.4 [150]. Importantly, this property is preserved during functionalization with other molecules, which makes it possible to form complex technological formulations without loss of stimulus sensitivity [151]. At present, the use of polymethacrylic acid as a component of regenerative and antibacterial agents, as well as in compositions with gold nanoparticles, copper nanoclusters, and silver, has been studied [152,153,154,155].

Recently, a method for the synthesis of nanosized cerium phosphate using polymethacrylic acid based on microwave synthesis was published. It is noteworthy that the experiment was carried out at two pH values: 1 and 11. At the same time, nanorods and almost spherical structures were naturally obtained, which indicates the absence of a negative influence of the polymer on the synthesis process. The most important result of this work can be considered the confirmation by X-ray photoelectron spectroscopy of the presence of both Ce^3+^ and Ce^4+^ in the structure of the obtained nano-objects, which indicates the preservation of redox activity with this approach. At the same time, it would be expected that the pH sensitivity of polymethacrylic acid could become a significant limitation in synthesis issues (which usually occur at rather extreme hydrogen index values and in the presence of ions), which was also refuted by this study [156].

Another example of the use of excipients in synthesis is the use of organic acids, the most popular of which at present is citric acid, as well as its salts. This is due to the fact that ionic agents, such as citrate, traditionally not only contribute to the creation of an optimal pH value, but also exert a stabilizing effect on nano-objects through adsorption of molecules on the particle surface and the formation of electrostatic interaction [157]. The addition of citrate during the synthesis of cerium orthophosphate exerts a specific effect. According to researchers, the molecular structure of the citric acid anion contributes to the formation of “flower-like” hierarchical structures due to the formation of new nucleation centers on already formed structures, playing the role of a chelating agent [73].

The literature also describes the use of cetyltrimethylammonium bromide (CTAB) as a stabilizer.

A notable feature is its addition in an amount equal to the precursor-the phosphate ion donor. Despite this, the results of Fourier transform infrared spectroscopy analysis indicate an undetectable amount of stabilizer in the final synthesis product. The researchers also noted that the stabilizing effect of CTAB does not exhibit a dose-dependent effect after reaching the minimum effective concentration that promotes the formation of ordered structures [73]. At the same time, it should be noted that when CTAB is used during hydrothermal synthesis, better results are observed for lanthanum phosphate rather than for cerium phosphate, the length of which again exceeds hundreds of micrometers, which does not solve the problem of obtaining nanorods of optimal size [158].

Thus, we can conclude that cetyltrimethylammonium bromide plays a significant role at the stage of cerium nanophosphate synthesis; however, to obtain stable products suitable for biomedical application, an additional excipient may be required to ensure nanoscale dimensions and system stability during long-term storage.

A comparative analysis of the excipients investigated as stabilizers for nanoscale cerium phosphate is presented in Table 2.

We can conclude that there is no universal methodology that allows the selection of optimal excipients for nanosized cerium phosphate at present. There is currently insufficient data in the literature regarding the effect of excipients on the physicochemical and pharmacological properties of nanoscale cerium phosphate. Key areas for future research include the evaluation of the pharmacokinetic profile of the resulting particles, as well as a comparative analysis of the activity of stabilized and unstabilized particles. However, at the moment, the using of biopolymers is the most studied approach and can be considered a promising direction in pharmaceutical development.

## 4. Possible Directions for the Application of Cerium Phosphate in Biomedicine

**Figure 4:**
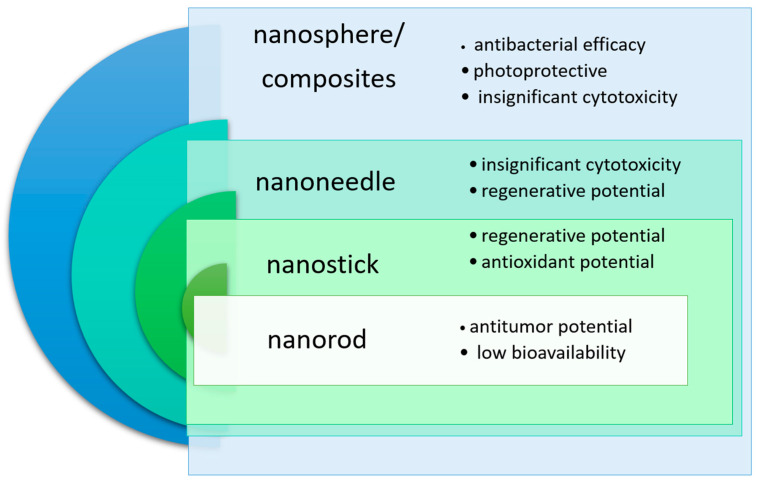
Schematic representation of the interplay between the morphology, activity and toxicity of nanoscale cerium phosphate.

Nanosized cerium orthophosphate has long remained in the shadow of cerium dioxide, which is better known in the international scientific community. Despite this, a number of studies report the possibility of using nanosized cerium phosphate in biomedicine. Although all these experiments are far from clinical studies, the unique properties of cerium phosphate associated with the features of its morphology determine its potential in various therapeutic fields. Basing on data from the current scientific literature [61,87], it can be assumed that there is a correlation between the morphology of nanoscale cerium phosphate, as well as its pharmacological activity and toxicological profile (Figure 4).

The antioxidant activity may be higher in nanoparticles with a larger surface area; however, their ability to penetrate cells remains a matter of debate. This makes these particles ideal for topical application, for example, as sunscreens. At the same time, regenerative, antibacterial and antitumor potential, as well as toxicity, may demonstrate a dependence on the smallest particle size, which determines their cellular permeability and biodistribution. However, data on this issue regarding rare-earth metals remain contradictory [56]. This problem is particularly acute in the case of cerium phosphate, for which data on pharmacological activity are limited; studies often lack standard pharmaceutical screening and do not provide detailed information not only on activity but also on particle morphology and size.

### 4.1. Redox Activity

Initially, the antioxidant activity of cerium is due to its ability to switch between two oxidation states of cerium (Ce^3+^ and Ce^4+^), which allows it to effectively neutralize reactive oxygen species such as superoxide (O_2_^−^), hydrogen peroxide (H_2_O_2_), and hydroxyl radical (•OH). Considering that cerium dioxide nanoparticles are best known for their pronounced antioxidant properties, confirmed in numerous studies [159,160,161], it became logical to consider whether nanosized cerium phosphate possesses such activity and what the features of manifestation of its redox activity are. As was already stated earlier, the uniqueness of cerium phosphate lies in how strongly the synthesis method, pH value, and composition of excipients influence its final physicochemical characteristics—up to the loss of signs of the presence of the reduced form [147].

In a study by Nicolini V. et al. [162] devoted to the evaluation of catalase-like activity of bioactive glasses initially containing cerium dioxide and P_2_O_5_, an interesting observation was made. The scientists found that in the glass matrix, P_2_O_5_ and cerium ions tend to interact, forming cerium phosphate in significant amounts, up to the complete non-detection of cerium dioxide. The assessment of antioxidant activity was carried out by titrimetric analysis using potassium permanganate and hydrogen peroxide (in two concentrations—0.1 M and 1 M). A dose-dependent catalase-like activity increasing over time was established. A significant color change was observed within the first 4 h. At the same time, the decrease in hydrogen peroxide concentration amounted to 64–90% and 17–35% for different concentrations over a one-week period [162]. This experiment, although it did not directly assess the properties of cerium phosphate, demonstrated its redox activity.

In another study, also initially devoted to nanoceria, an analysis was conducted of the influence of various anions (chlorides, phosphates, sulfates) on the redox activity of cerium in general and on its ability to exert DNA-protective action in particular. The assessment of the influence of phosphate was carried out at pH 4.7 and 7.4. According to the conclusion of the experimenters, in the presence of phosphate, cerium did not demonstrate a protective effect. This was explained by the fact that phosphate on the particle surface disrupts the Ce^3+^/Ce^4+^ redox cycle [163]. Similar results were obtained in another publication evaluating the relationship between anions and the ability of cerium to protect DNA from damage by 30% hydrogen peroxide using square-wave voltammetry [164]. In the context of the present review, these studies have a significant limitation: it is unknown how justified it is to fully extrapolate these data to nanosized cerium phosphate.

In contrast, when evaluating redox activity by the chemiluminescence method measured in the Fenton reaction, it was revealed that nanosized cerium phosphate possesses a dose-dependent antioxidant effect. At the same time, the value of the light sum integral for cerium was 11 times higher than that for the reference antioxidant (ascorbic acid). This pattern was observed throughout the entire concentration range (from 10^−5^ M to 10^−2^ M). It should be noted that the highest concentration can potentially exert a toxic effect, and the lowest may demonstrate an insufficient effect under in vivo conditions [61].

Thus, CePO_4_ should be regarded as a redox platform in which the redox behavior is determined not only by the valence state of cerium but also by the phosphate matrix, which stabilizes ionic and surface centers. Unlike CeO_2_, where functional activity is largely associated with oxygen non-stoichiometry and the Ce^3+^/Ce^4+^ cycle on the surface of oxide nanocrystals, in CePO_4_, the phosphate framework stabilizes cerium and modulates the accessibility of redox centers. This alters the accessibility of redox centers and can either reduce or, conversely, selectively modulate catalytic reactivity depending on morphology, crystallinity, and degree of hydration. In particular, this can lead to lower photocatalytic activity while maintaining a pronounced photoprotective and antioxidant potential. This allows CePO_4_ to be considered a nanomaterial with more selective surface chemistry (compared to CeO_2_ nanoparticles), suitable for tasks that require a balance between catalytic activity and biocompatibility.

Apparently, in order to make an unambiguous conclusion about redox activity of cerium phosphate, additional studies using carefully validated methodologies are required, which could not only confirm or refute the effect, but also identify factors that influence the redox properties of cerium phosphate and are capable of determining its applicability in medical practice. An important step toward addressing this issue may be a comprehensive study involving nanoscale cerium phosphate, in which IC50/EC50 values are determined in accordance with FDA recommendations.

### 4.2. Regenerative and Antibacterial Properties

Most likely, it is precisely in connection with antioxidant properties that cerium dioxide nanoparticles demonstrate regenerative properties repeatedly confirmed in experiments both using cell cultures and in vivo [165,166,167,168,169]. Information on the possibility of using cerium phosphate as a regenerative agent is limited and is presented only in a few scientific papers.

In particular, in 2024 a study was published in which terbium-doped cerium phosphate was part of a gel that also included gelatin, methacrylic acid, and manganese glass. The scientists established a reduction in oxidative stress and intensification of proliferation and differentiation of fibroblast cells under in vitro conditions. Regenerative properties were also confirmed in vivo, which makes this composition a promising candidate for work with chronic wound defects [170].

That results were confirmed in 2025 [60,61]. Based on work with mesenchymal stem cells (MSCs), it was found that samples of nanosized cerium phosphate stimulated proliferation for 48 h. Similar results were obtained when working with cultures of human fibroblast (BJ hTERT) and keratinocyte cells (HaCaT) [61]. In one study [60], a pronounced stimulating biological effect of nanosized cerium phosphate compounds, including methylcellulose-based nanocomposites, on cell cultures involved in regeneration was established, which determines the potential for creating an optimal medical device containing cerium phosphate nanoparticles for healing wounds of various origins.

Based on the analysis of the regenerative activity of cerium phosphate, we can conclude that the information is currently limited to in vitro studies conducted using cell cultures. Nevertheless, the above-mentioned studies are completely new, and therefore they may increase the interest of the international scientific and medical community in conducting further experiments and developing the topic.

In addition to the problem of wound healing itself, chronic wounds are associated with the risk of developing infectious complications. As a result, the possibility of using cerium phosphate as an antibacterial agent should be considered. At present, studies of phosphate without other pharmacologically active substances that could influence the result have not been published; however, for the combination of phosphate and cerium dioxide, high antibacterial activity has been described against Gram-negative (using *Salmonella typhimurium* and *Escherichia coli* as examples) and Gram-positive bacteria (such as *Staphylococcus aureus* and *Bacillus cereus*) without signs of cytotoxicity toward HeLa cervical cancer cells and Vero monkey kidney cells [88]. The latest publications also indicate the presence of an antifungal effect when used jointly with silver nanoparticles [171]. With regard to antibacterial properties, we once again encounter a lack of information in peer-reviewed scientific publications; however, it should be noted that in recent years interest in this topic in the context of cerium phosphate has been increasing.

### 4.3. Photoprotective and Antitumor Properties

One of the unique properties of cerium phosphate discussed in the scientific literature is its ability to exert a protective effect against ultraviolet radiation [172], due to its ability to absorb in the UV region of the spectrum and its comparatively low catalytic activity, which is significantly less applicable to cerium dioxide [173,174]. At the same time, it was established that cerium phosphate obtained by the hydrothermal method contains a smaller amount of other cerium derivatives and is a more effective ultraviolet absorber [175].

According to the results of studies published in 2024, it was revealed that the photocatalytic activity of cerium compounds ranges from 0.5 to 0.1 of the activity of titanium dioxide, which significantly reduces the risk of interaction with organic components of sunscreen cosmetic and cosmeceutical products [176]. At the same time, the SPF (Formula (1)) ranges from 1 to 2.9 and exceeds that of titanium-containing compositions [176,177].

(1) $$SPF=\frac{MED_{p}}{MED_{u}}$$

*MED_p_*—Minimal erythemal dose according to ISO 24444:2019.

*MED_u_*—Minimal erythemal dose on unprotected skin according to ISO 24444:2019 [178].

In a study using mesenchymal stem cells and mouse fibroblasts L929, it was established that the investigated samples do not exhibit cytotoxic properties [176]. At the same time, some publications indicate an even higher SPF level (30.04–48.52) at a concentration of 100 µg/mL [179]. Notably, the ability to absorb ultraviolet radiation is retained even in the complete absence of auxiliary substances [180]. Taking into account the totality of these factors, as well as considering better organoleptic properties and greater physicochemical stability compared to standard inorganic UV filters, it was suggested that cerium phosphate may be applied in larger quantities, which would provide an even higher level of protection [181].

Based on this, it can be concluded that nanosized cerium phosphate should be considered an effective and safe agent protecting against the adverse effects of ultraviolet radiation, potentially surpassing existing inorganic UV filters. It is quite possible that cerium phosphate may one day become an effective means for the prevention of melanoma and other tumor diseases.

In addition to its preventive role with respect to certain malignant neoplasms, it is advisable to consider the possibility of using nanosized cerium phosphate as an antitumor agent. At present, such a possibility has been demonstrated in the work of Ge Y.W. et al., which consisted of an in vitro experiment using photothermal tumor therapy. Notably, despite the significant size of the nanorods (diameter 50 nm, length approximately 5 µm), a cytotoxic effect against mouse RAW264.7 cells was identified, indicating that significant length is not an obstacle to penetration through cellular membranes. At the same time, it was revealed that nanosized cerium phosphate not only did not exert an effect on normal human bone marrow mesenchymal stem cells hBMSC and mouse osteoblasts MC3T3-E1, but also promoted their proliferation, probably by activating the BMP-2/Smad signaling pathway [182].

### 4.4. Toxicity

The toxicological profile represents a significant challenge for the application of rare-earth metals in biomedicine, which is associated with their ability to accumulate in the liver and spleen [52]. At the same time this issue is likely most acute, in the context of recent developments, for nanoscale cerium phosphate, for which data on toxicity remain quite limited. The available data have been obtained predominantly from in vitro experiments.

Most studies have assessed the risk of acute cytotoxicity using cell cultures. The MTT assay with HeLa cervical cancer cells has been most commonly employed, and no signs of adverse effects were identified in any of these studies [87,88,183]. Similar results were obtained in studies involving human fibroblasts and keratinocytes [61]. In addition, Zhang F. and Wong S.S. proposed a putative pharmacokinetic behavior of the particles, according to which cerium phosphate enters cells via diffusion and receptor-mediated endocytosis, both of which exhibit temperature dependence [183].

However, in a study dated 2009, the authors draw attention to an important feature of testing cerium phosphate: prior to performing the MTT assay, nanoscale cerium phosphate was subjected to additional ultrasonic treatment for 1 h. As a result, extrapolating the obtained results to larger nanorods and nanowires may be inappropriate [183].

Another limitation is represented by data indicating a decrease in proliferative activity after 48 h when cerium phosphate is applied at concentrations of 10^−2^ M and higher, as demonstrated in experiments using mesenchymal stem cells [61]. Based on this, it can be concluded that the effects of nanoscale cerium phosphate at the cellular level require thorough investigation under conditions as close as possible to real conditions, as well as careful dose selection.

In turn, the study of the toxicological profile of nanoscale cerium phosphate is likely of critical importance also in the context of nanoscale cerium dioxide, which has the ability to transform into phosphate [184]. This phenomenon has been observed in biological systems as well, as described by Zhang P. et al. According to their observations, nanoceria is capable of forming rod-like phosphate crystals in the intercellular space of plants, which prompts investigation of similar processes in animal organisms [185]. At the same time, it cannot be excluded that cerium phosphate nanoparticles may exhibit higher biocompatibility as was demonstrated, for example, in a comparative study of phytotoxicity for *Lactuca sativa Linn*., where oxide and phosphate with similar morphology were compared; this was explained by the greater biochemical inertness of the latter [186]. Interesting observations were also made in in vivo experiments. Yokel R.A. et al. evaluated toxicokinetics and oxidative stress following intraperitoneal administration of 10 mg/kg nanoceria in female mice C57BL/6 and BALB/c [187]. In this study, the formation of cerium phosphate nanoneedles in the organism was also recorded. In particular, the formation of nanoscale phosphate was observed in the liver and spleen as early as 30 min after injection and persisted after 24 h. The response to administration was accompanied by an increase in caspase-1 levels in the liver after 6 h and accumulation of ferritin in lysosomes near the nano-objects. The most pronounced effect was observed in BALB/c mice [187], which may be due to differences in immune response formation and iron homeostasis in these strains [188].

The accumulation of cerium phosphate is described in more detail in the work by Graham U.M. and et al. [189]. According to the study design, nanoceria was administered to male Sprague Dawley rats at a dose of 85 mg/kg. Nanoparticles and their degradation products were detected after 90 days. It was established that the formation of cerium phosphate nanoneedles occurred predominantly in the white pulp of the spleen, forming agglomerates up to several hundred nanometers in length. According to the authors the toxicokinetics of nanoparticles is a complex and multifaceted phenomenon that is not limited to accumulation and subsequent elimination, but is accompanied by a complex chain of bioprocessing processes, resulting in the formation of multiple generations of derivatives [189].

Since the biological activity (both therapeutic and toxic) of nanosized cerium phosphate is a complex and poorly understood phenomenon, we have summarized the main findings of in vitro studies using cell cultures and in vivo studies in Table 3.

Thus, it can be concluded that, regardless of the actual prospects for the clinical implementation of nanoscale cerium phosphate, comprehensive toxicological studies are required. This is due to the ability of nanoscale cerium dioxide—already very close to medical application—to transform into phosphate nanoneedles under in vivo conditions. In addition, to further advance the field and accelerate the implementation of cerium-containing nanomaterial-based medical products, it may be recommended that researchers perform biocompatibility assessments in accordance with ISO 10993 and conduct risk assessment based on OECD guidelines.

## 5. Discussion

In general, nanosized cerium orthophosphate represents a potentially promising antioxidant [61,162] and regenerative agent [60,61,170] with a wide range of potential applications in biomedicine. At the same time considerable attention from researchers is focused on studying the ability of cerium phosphate nanoparticles to protect against exposure to ultraviolet radiation [174,175,176,177,178,179]. Photoprotective properties are of immense importance in preventive medicine, as ultraviolet radiation significantly increases the risk of melanoma and other malignant skin neoplasms [190]. It should be noted that this particular direction is currently the most developed.

Basing on the presented data, we can conclude that cerium nanocomposites and nanoneedles are the most extensively studied in terms of therapeutic and adverse effects [88,176,187,189]. However, potentially clinically significant in vivo studies have been conducted only for nanoneedles and modified nanorods [181,187,189]. The results obtained allow us to suggest that the most promising areas of application are as regenerative and antioxidant agents [61,182,189]. At the same time, the possibility of using nanosized cerium phosphate as a cosmeceutical photoprotective agent should not be excluded [174,176]. The lack of in vivo studies in the context of topical cosmetic products is not a critical obstacle, which is consistent with current trends away from testing cosmetics on animals. It should be noted, however, that although the current data on the toxicological profile of nanoscale cerium phosphate across all the morphological variants presented are encouraging, we have identified a significant limitation. In particular, there is currently a lack of sufficient data on biodistribution, long-term accumulation, immunogenicity, and methods of excretion. The currently available data on organ-specific toxicity point to a negative effect on spleen and, to a lesser extent, liver parenchyma, which is likely due to the relative inertness of cerium phosphate and its low solubility [187,189]. However, concerns regarding the potential neurotoxicity of nanoscale rare-earth metals have not been confirmed [52]. Furthermore, a crucial step required for clinical implementation must be the conduct of a comprehensive toxicological study of chronic toxicity, the determination of key parameters (LD50, LD100, LD0, CL0, etc.), as well as studies on the effects of cerium phosphate via various routes of administration, which would help to fill the most significant gaps in information for the international scientific community.

However, there are currently significant constraints that require resolution before the use of nanoscale cerium phosphate in actual clinical practice can become a reality. In particular, a major challenge is the lack of a standardized methodological approach to the synthesis of nanoscale cerium phosphate. One possible approach to solving this problem is the using of predictive synthesis. Predictive synthesis of nanosized cerium phosphate should be described as a sequential adjustment of morphology through a set of interrelated parameters, rather than as the selection of a single “optimal” condition. According to the literature, it is pH, temperature, the nature of the precursors, and the ratio of reagents that most strongly determine the phase composition, particle size, degree of aggregation, and final form—ranging from nanospheres to nanoneedles [68,72,76,90]. It is most productive to consider CePO_4_ synthesis as a hierarchical growth control scheme. First, the chemical window for phase formation is set by selecting precursors and reagent ratios; then, the rates of nucleation and crystallization are controlled via pH and temperature; and in the final stage, the solvent and stabilizing polymers regulate growth, aggregation, and the spatial organization of the particles. Control of the impurity composition is no less important. Residual precursor ions, by-product cerium-containing phases, traces of solvents, surfactants, stabilizers, and products of partial material transformation can determine both the effectiveness and the safety profile of the composition under study. Therefore, CePO_4_ requires comprehensive physicochemical characterization, including confirmation of phase composition, elemental profile, surface chemistry, and residual impurity content using modern analytical methods.

Storage stability remains a separate challenge. The long-term stability of the product under various storage conditions must be documented prior to the start of preclinical trials, and even more so before clinical trials. The existing data for CePO_4_ remains extremely limited. For potential biomedical applications, it is not sufficient to demonstrate the properties of a freshly prepared sample. It is necessary to confirm the preservation of particle size, zeta potential, degree of aggregation, valence state of cerium, colloidal stability, and functional activity during storage under relevant conditions. The lack of such data complicates both the interpretation of preclinical results and the transition to scale-up and the development of a drug formulation or medical device. Directly related to this is the issue of batch-to-batch reproducibility of the physicochemical properties of nanoparticles. A systematic multi-laboratory study covering 46 batches of OECD priority nanomaterials, including cerium dioxide, showed that even when using the same synthesis protocol, significant variability in particle size, shape, surface charge, and aggregation state is observed [191].

Furthermore, working with rare-earth metals entails certain risks in terms of ensuring production stability and safety, as well as compliance with GMP requirements. In particular, to achieve the required reproducibility in accordance with GMP principles, strict standardization of key parameters is necessary: the pH of the reaction medium, temperature, synthesis time, reagent ratios, precursor concentrations, and the regimen for adding stabilizing agents. For nanoscale systems, it is fundamentally important to demonstrate that independent batches produced using the same protocol possess comparable critical quality indicators and elicit a similar biological response. Without this, even positive results from individual experiments remain difficult to reproduce and do not allow for a reliable assessment of the technology’s prospects.

Another significant limitation is the lack of information on many toxicological characteristics. In particular, a major gap is the lack of data on cumulative toxicity, chronic toxicity, genotoxicity, hemocompatibility, routes of elimination, and immunogenic potential. One possible way of improving safety is to select an optimal formulation, likely containing biocompatible and biodegradable polymers, which could ensure targeted delivery, sustained release of the nanoparticle, and reduce the risk of side effects while maintaining the primary pharmacological effect [56,135,141]. The most promising polymer at present is 10% polyvinylpyrrolidone with molecular weight of 360,000, as it enables the production of aggregately stable nanoneedles [107], while the literature already describes the preservation of antioxidant properties, as well as the cytoprotective effect of the combination of cerium and polyvinylpyrrolidone [56]. Nanoneedles are currently the most extensively studied morphological form of nanoscale cerium phosphate. Antioxidant properties have been identified for this form, and the most comprehensive toxicity data have been obtained, making nanoneedles a significant candidate for pharmaceutical development [187,189]. In addition to the excipients already discussed in this article, other polymers that have already proven themselves as stabilizers may be considered. Examples of such biopolymers include hyaluronic acid or dextran [56]. It is quite possible that machine learning—specifically artificial intelligence capable of identifying hidden patterns—could be employed to accelerate the screening of potential formulations at minimal cost. It can be particularly important given the lack of information on optimal combinations and the absence of a methodology for producing a pharmaceutical formulation containing cerium phosphate [192].

At the same time, the most significant problem is that the small number of in vivo studies, as well as the complete absence of clinical studies, form a vicious circle in which, due to the insufficient number of published materials, a potentially promising therapeutic agent becomes even less studied and discussed. Thus, future research should be aimed at conducting a full cycle of experiments, from validated in vitro experiments using chemical models and cell culture studies to in vivo investigations, in order to achieve a truly objective assessment of the use of nanosized cerium phosphate as an agent for biomedical application.

## 6. Conclusions

Based on the results of the conducted review, it can be concluded that nanosized cerium phosphate may be a promising candidate for the development of agents for biomedical application. A number of synthesis methods, such as hydrothermal synthesis and chemical precipitation, are well studied, and their validity has been confirmed by numerous studies. It is also necessary to note the fact that by regulating reaction conditions such as pH, temperature, and the use of microwave radiation, it is possible to achieve a specific morphology that will best correspond to the particular objectives of researchers.

At present, the most actively discussed possibility is the development of cerium-containing agents for the production of medical devices possessing protective properties against ultraviolet radiation, which may become an important step in the prevention of such an oncological disease as melanoma. There are other potential CePO_4_ application areas, such as use as an antioxidant or regenerative agent. However, the issue of creating medicines containing nanosized cerium phosphate is at an early stage of development and, consequently, requires many further studies both in vitro and in vivo.

The key requirements for the actual translational advancement of cerium phosphate remain standardized synthesis, impurity control, confirmation of storage stability, interbatch comparability, and comprehensive in vitro and in vivo toxicological assessment. Therefore, further development of this field should be focused not only on the search for new biomedical applications of CePO_4_, but also on the development of a comprehensive research strategy that integrates rational synthesis design, multi-level physicochemical characterization, stability assessment, standardization of critical quality parameters, and extensive preclinical toxicological validation.

## Figures and Tables

**Table 1:** Key characteristics of synthesis methods for nanosized cerium phosphate (“+” indicates acceptable reproducibility, “++” indicates high reproducibility, “-” means there are not enough data to draw conclusions).

Method	Precursors	Size, nm	Morphology	Specific Conditions (t, °C)	Process Duration, h	Zeta Potential	Reproducibility	Advantages	Limitations	Sources
Hydrothermal	CeCl_3_ and Na_3_PO_4_; Ce(NO_3_)_3_ and H_3_PO_4_; Ce(NO_3_)_3_ and (NH_4_)_2_HPO_4_	5–50 in diameter; length 200–1000	Nanowires; nanorods; spherical; hexagonal and monoclinic	100–200	≥14–36	No data	+	The ability to control morphology by adjusting parameters	Potentially unsuitable for use as an antibacterial or regenerative agent	[70,71,72,73,74]
Chemical precipitation	Ce(NO_3_)_3_ and NH_4_H_2_PO_4_	2–10 in diameter; length 30–50	Nanospheres; nanoneedles	200–800	≥26	No data	++	The smallest size of nano-objects, presumably offering the best cellular permeability; the formation of porous spherical structures, which may indirectly indicate the highest redox activity	Particle agglomeration; labor-intensity	[61,76]
Microwave	Ce(NO_3_)_3_ and Na_3_PO_4_	8–50 nm in diameter; length up to 3000	Nanorods; nanospheres	130–180	24	No data	+	Low risk of the formation of synthesis by-products	Potentially unsuitable for use as an antibacterial or regenerative agent; particle agglomeration; expensive equipment	[68,79]
Sol–gel	CeO_2_ and H_3_PO_4_; CeCl_3_ and H_3_PO_4_	5–60 nm in diameter; length over 1000	Nanoribbons; nanofibers	≥900	≥240	No data	-	The production of organoleptically appealing products that are potentially suitable for use as cosmetics and medical devices	Potentially unsuitable for use as an antibacterial or regenerative agent; the longest duration; exposure to extreme temperatures	[69,81,82]
Green synthesis	Ce(NO_3_)_3_ and H_3_PO_4_; Ce(NO_3_)_3_ and marine *Sinularia polydactyla* extract; Ce(NO_3_)_3_ and *Artocarpus heterophyllus* aqueous leaf extract	4–8 nm in diameter; length 25.81–200	Nanowires; urchin-like structures; monoclinic	Room temperature; 500–600	2–3; 24	No data	-	An environmentally friendly method; the plenty of scope for innovation and various modifications	The difficulty of controlling and standardizing the synthesis	[87,88]

**Table 2:** Key characteristics of excipients for nanosized cerium phosphate synthesis.

Excipient	Colloidal Stability	Biodistribution	Functionality	Therapeutic Efficacy	Sources
PVP	No data	No data	The ability to produce bundles or individual rods depending on molecular weight	Antioxidant activity	[70,107]
PEG	No data	No data	The formation of soft hexagonal “coral-like” structures	Redox activity	[90]
Pluronic^®^	No data	No data	Improved reproducibility of synthesis, uniformity of size	Pro-oxidant activity	[145,147]
PMAA	No data	No data	The ability to synthesize over a wide pH range	Redox activity	[156]
Citric acid	No data	No data	The formation of “flower-like” hierarchical structures	No data	[73]
CTAB	Identified	No data	No data	No data	[73]

**Table 3:** The biological activity of nanosized cerium phosphate.

Morphology	Dose	Activity	Toxicity Profile	Methods	Sources
CeO_2_–CePO_4_ nanocomposites	0.1–3 mg/mL	Antibacterial	Non–toxic up to 3 mg/mL	In vitro; *B. cereus*, *S. typhimurium*, *E. coli*, *S. aureus*; HeLa, Vero	[88]
	0.125–1 mg/mL	Photoprotective	Low toxicity	In vitro; human MSCs, NCTC L929 mouse fibroblasts	[176]
No data	0.125–1 mg/mL	Photoprotective, but minimal in the form of monazite	Low toxicity	In vitro; human MSCs, NCTC L929 mouse fibroblasts	[176]
Nanospheres	50–200 μg mL^−1^	Photoprotective	Low toxicity	In vitro; HaCaT	[174]
Tb-doped nanowires	0.1–0.5 mg/mL	Redox, photoluminescence	Low toxicity	In vitro; HeLa	[183]
Nanosticks	10^−2^ to 10^−5^ M	Regenerative, antioxidant, an increase in cellular metabolism of 1.11–1.29 times	Cumulative exposure to a concentration of 10^−2^ M for more than 48 h had a negative effect on the proliferative activity of MSCs. No cytotoxic effect	In vitro; human MSCs, HaCaT, BJ hTERT	[61]
Needle-like clusters	2 mg/mL	Redox	Accumulation on the root epidermis and in the intercellular spaces	Hydroponic cucumber plants	[185]
Nanorods	2 mg/mL	No effect	No cytotoxicity and no adverse effect on enzymatic activity	*Lactuca sativa* Linn	[186]
Graphene-modified Nanorods	80 mg/mL	Bone tissue regeneration, tumor cell apoptosis, regenerative for normal cells	No cytotoxicity	In vitro; MC3T3-E1, RAW264.7, and MDA-MB-231 cells; In vivo, ex vivo; mice and Sprague Dawley rats	[182]
Nanoneedles	10 mg/kg of CeO_2_, which is converted in situ into CePO_4_	No data	Acute toxicity (0.5–24 h) includes activation of caspase-1 in liver, decreased hepatic vacuolization, increased spleen lymphoid white pulp cell density, elevated ferritin levels	In vivo, ex vivo; BALB/c and C57BL/6 mice	[187]
	85 mg/kg of CeO_2_, which is converted in situ into CePO_4_	Antioxidant	Subacute toxicity (90 days) included: the formation of granulomas in liver and spleen. Cytoplasmic agglomerates containing Ce were observed in macrophages, with a higher prevalence in white pulp. The cerium content in liver between 30 and 90 days was accompanied by no reduction or even an increase in its content in spleen (2800 μg/g), which exceeds the values for liver (300 μg/g)	In vivo, ex vivo; Sprague Dawley rats	[189]

## Data Availability

Data is contained within the article.

## Abbreviations

The following abbreviations are used in this manuscript:

PVP	polyvinylpyrrolidone
PEG	polyethylene glycol
PMAA	polymethacrylic acid
CTAB	cetyltrimethylammonium bromide
ROS	reactive oxygen species
SPF	sun protection factor
UV	ultraviolet
